# The Effect of Vitamin D Supplementation Post COVID-19 Infection and Related Outcomes: A Systematic Review and Meta-Analysis

**DOI:** 10.3390/nu16223794

**Published:** 2024-11-05

**Authors:** Marina Sartini, Filippo Del Puente, Alessio Carbone, Elisa Schinca, Gianluca Ottria, Chiara Dupont, Carolina Piccinini, Martino Oliva, Maria Luisa Cristina

**Affiliations:** 1 Operating Unit Hospital Hygiene, Galliera Hospital, 16128 Genoa, Italy; alessio.carbone@galliera.it (A.C.); elisa.schinca@unige.it (E.S.); gianluca.ottria@unige.it (G.O.); martino.oliva@galliera.it (M.O.); maria.luisa.cristina@galliera.it (M.L.C.); 2 Department of Health Sciences, University of Genoa, 16132 Genoa, Italy; lioa@unige.it (C.D.); carolina.piccinini@libero.it (C.P.); 3 Department of Infectious Diseases, Galliera Hospital, 16128 Genoa, Italy; filippo.del.puente@galliera.it

**Keywords:** vitamin D, COVID-19, mortality, intensive care unit

## Abstract

Background: Vitamin D’s role in COVID-19 management remains controversial. This meta-analysis aimed to evaluate the efficacy of vitamin D supplementation in patients with SARS-CoV-2 infection, focusing on mortality, intensive care unit (ICU) admissions, intubation rates, and hospital length of stay (LOS). Methods: A systematic review of PubMed/MEDLINE, Scopus, Cochrane, and Google Scholar databases was conducted. Randomized controlled trials (RCTs) and analytical studies investigating vitamin D supplementation in COVID-19 patients were included. The meta-analysis was performed using STATA MP 18.5, employing random-effect or fixed-effect models based on heterogeneity. Results: Twenty-nine studies (twenty-one RCTs, eight analytical) were analyzed. Vitamin D supplementation significantly reduced ICU admissions (OR = 0.55, 95% CI: 0.37 to 0.79) in RCTs and analytical studies (OR = 0.35, 95% CI: 0.18 to 0.66). Intubation rates were significantly reduced in RCTs (OR = 0.50, 95% CI: 0.27 to 0.92). Mortality reduction was significant in analytical studies (OR = 0.45, 95% CI: 0.24 to 0.86) but not in RCTs (OR = 0.80, 95% CI: 0.61 to 1.04). Subgroup analyses revealed more pronounced effects in older patients and severe COVID-19 cases. LOS showed a non-significant reduction (mean difference = −0.62 days, 95% CI: −1.41 to 0.18). Conclusions: This meta-analysis suggests potential benefits of vitamin D supplementation in COVID-19 patients, particularly in reducing ICU admissions. However, the evidence varies across outcomes and patient subgroups. Discrepancies between RCTs and analytical studies highlight the need for further large-scale, well-designed trials accounting for baseline vitamin D status, standardized supplementation protocols, and patient characteristics to inform clinical guidelines for vitamin D use in COVID-19 management.

## 1. Introduction

Vitamin D is widely recognized for its essential role in bone metabolism; however, its potential implications extend far beyond skeletal health [1,2]. Recent research showed the broader functions of vitamin D, particularly through its interaction with the vitamin D receptor (VDR), a ligand-dependent transcription regulator [3,4,5,6]. This receptor, which is expressed by nearly all nucleated human cells, influences the expression of approximately 4% of the human genome. Consequently, the effects of vitamin D encompass a diverse array of physiological cellular processes via induction or repression of gene transcription [6,7,8].

Over the years, numerous studies have explored the extraskeletal roles of vitamin D [9,10], which has been theorized to be implicated in muscle metabolism [11,12], cancer incidence and prevention [13,14], diabetes prevention [15], cardiovascular function [16], and immune response to both self-antigens and pathogens [17,18,19], such as Mycobacterium tuberculosis [20]. In the infectious disease field, vitamin D supplementation has been shown to promote sputum conversion in patients with smear-positive tuberculosis, although it seems to not significantly impact mortality or primary infection prevention [21,22,23]. While evidence for a reduction in the incidence of upper respiratory infections (URIs) with vitamin D supplementation is lacking [24,25], some benefits have been observed in reducing exacerbations of chronic obstructive pulmonary disease (COPD) in patients with severe vitamin D deficiency (levels below 10 ng/mL) [26]. Additionally complicating analysis, specific thresholds for vitamin D levels (and consequently, appropriate dosages) have not yet been established for extraskeletal health [27].

The onset of the SARS-CoV-2 pandemic has prompted further investigation into the potential role of vitamin D in infectious disease management [28,29,30,31]. Although definitive evidence is still lacking, preliminary data suggest that vitamin D supplementation may reduce the risk or severity of COVID-19 in some patients [32].

The mechanisms through which vitamin D may influence COVID-19 outcomes are multifaceted. Vitamin D exerts immunomodulatory effects through several pathways: (1) regulation of pro-inflammatory cytokines, particularly reducing IL-6 and TNF-α levels; (2) enhancement in antimicrobial peptide production, including cathelicidin and β-defensins; (3) improvement in epithelial barrier integrity; and (4) modulation of T-cell responses and enhancement in regulatory T-cell function. These mechanisms may be particularly relevant in preventing the hyperinflammatory state characteristic of severe COVID-19.

This meta-analysis aims to evaluate whether vitamin D can serve as a protective agent in patients with SARS-CoV-2 infection, focusing on its therapeutic role rather than a prophylactic one. The goal is to assess the efficacy of vitamin D within a comprehensive treatment regimen that includes antiviral, corticosteroid, and anticoagulant therapies aligned with current clinical guidelines.

Through this meta-analysis, we seek to provide a clearer understanding of the potential benefits of vitamin D in the management of SARS-CoV-2 infections, contributing to the optimization of treatment protocols and patient outcomes in the ongoing fight against COVID-19.

## 2. Materials and Methods

This systematic review and meta-analysis were conducted in accordance with the Preferred Reporting Items for Systematic Reviews and Meta-analyses (PRISMA) guidelines, ensuring adherence to current standards for reporting systematic reviews [33] (Supplementary Materials File S1). The research question was formulated using the PICO framework. The population of interest comprised patients, particularly those in the ICU, who received vitamin D supplementation following a COVID-19 diagnosis. The primary outcomes evaluated were mortality, ICU admissions, intubation, and the length of hospital stay (LOS). The study protocol was registered in the PROSPERO database (registration number CRD42023469826).

### 2.1. Data Sources and Search Strategy

An exhaustive search was carried out using a combination of keywords (“COVID-19” OR “SARS- CoV-2” OR “coronavirus” OR “2019-nCoV”) AND (“vitamin D” OR “cholecalciferol” OR “calcitriol”) across the PubMed/MEDLINE, Scopus, Cochrane, and Google Scholar databases up to February 2024. The National Center for Biotechnology Information (NCBI) nomenclature and guidelines were followed when using Medical Subject Heading (MeSH) terminology, with the addition of wildcards when necessary.

### 2.2. Studies Selection

The inclusion criteria consisted of (1) studies providing relevant quantitative data on the association between Vit D supplementation after a COVID-19 diagnosis and significant clinical outcomes and (2) randomized controlled trials (RCTs), cohort studies, or quasi-experimental research designs.

The exclusion criteria included: (1) studies not directly related to the search query; (2) studies lacking sufficient data on the impact of vitamin D supplementation post-COVID-19 diagnosis and relevant outcomes; (3) studies not adhering to the PICOS criteria, defined as:

- P: Patients, including ICU patients, diagnosed with COVID-19;
- I: Patients receiving vitamin D supplementation post COVID-19 diagnosis;
- C: Patients receiving standard treatment, a lower dose of vitamin D, no treatment, or a placebo;
- O: outcome including mortality, ICU admission, intubation, and hospital length of stay (LOS) associated with vitamin D intake;
- S: RCTs, cohort studies, and quasi-experimental studies.

Studies that did not satisfy these criteria were excluded. No restrictions were applied regarding the publication date or language. Detailed information on the search strategy can be found in Table 1.

### 2.3. Data Extraction and Risk of Bias Assessment

Four authors independently conducted the initial literature screening. Any discrepancies that emerged were resolved through discussion until a consensus was achieved. After the full-text review, studies selected for inclusion proceeded to data extraction.

Data for the meta-analysis were collected from the reviewed studies using a standardized extraction form. Essential details gathered included the first author’s last name, year and country of publication, study design, counts of deaths, ICU admissions, intubations, hospital length of stay, enrollment period, the severity of the patient’s condition related to COVID-19, baseline vitamin D status, age and sex of participants, and comparison type in each study. The comparisons included groups such as vitamin D supplementation versus no treatment, high-dose versus low-dose vitamin D supplementation, vitamin D versus placebo, intervention duration, the amount of vitamin D given to the treated group (and to the control group if available), number of participants enrolled, and distributions across subgroups.

Effect measures calculated comprised Odds Ratios (ORs) with 95% confidence intervals (CIs) for binary outcomes and Mean Difference for continuous outcomes, such as the duration of hospital stay. For studies reporting continuous outcomes in terms of medians and interquartile ranges, the corresponding mean and standard deviation were estimated using the methods provided by Luo et al. [34] and Wan et al. [35]. Quality and potential bias in the studies were assessed independently by four researchers, employing tools tailored to each study type. For RCTs, the National Institutes of Health (NIH) quality assessment tool for controlled intervention studies was utilized, while the NIH quality assessment tool for observational cohort and cross-sectional studies was applied to cohort and cross-sectional studies [36]. The JBI critical appraisal tool for quasi-experimental studies was used to assess quasi-experimental studies [37].

During the quality assessment, researchers concluded that for RCTs, the criterion “Were the outcome assessors blinded to participants’ group allocation?” had limited applicability. This was due to the fact that, in typical medical practice, outcomes are usually unaffected solely by vitamin D administration. As such, it could reasonably be assumed that clinical decision making in the RCTs was not significantly impacted by the participant assignment to vitamin D supplementation groups. Any residual disagreements were addressed by reaching a consensus.

**Figure 1:**
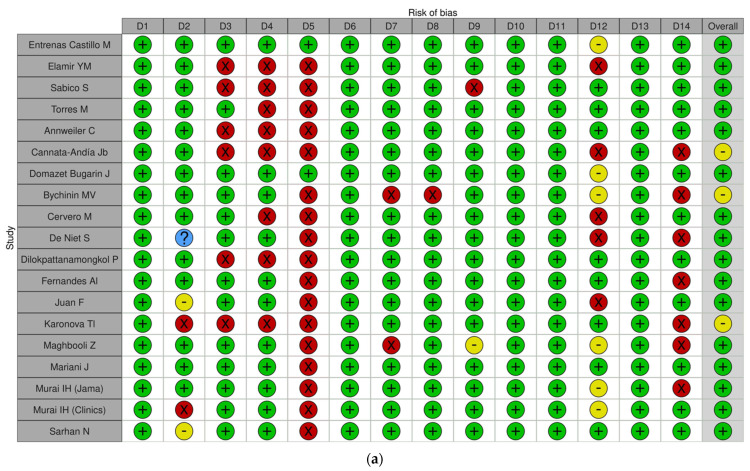

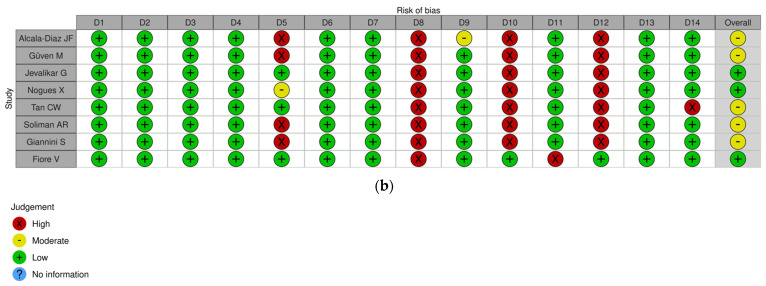
Traffic light plots of Risk of Bias for RCT (**a**) and for analytical studies (**b**). (**a**) D1: Was the study described as randomized, a randomized trial, a randomized clinical trial, or an RCT? D2: Was the method of randomization adequate (i.e., use of randomly generated assignment)? D3: Was the treatment allocation concealed (so that assignments could not be predicted)? D4: Were study participants and providers blinded to treatment group assignment? D5: Were the people assessing the outcomes blinded to the participants’ group assignments? D6: Were the groups similar at baseline in terms of important characteristics that could affect outcomes (e.g., demographics, risk factors, comorbid conditions)? D7: Was the overall drop-out rate from the study at the endpoint 20% or lower than the number allocated to treatment? D8: Was the differential drop-out rate (between treatment groups) at endpoint 15 percentage points or lower? D9: Was there a high adherence to the intervention protocols for each treatment group? D10: Were other interventions avoided or similar in the groups (e.g., similar background treatments)? D11: Were outcomes assessed using valid and reliable measures implemented consistently across all study participants? D12: Did the authors report that the sample size was sufficiently large to detect a difference in the main outcome between groups with at least 80% power? D13: Were outcomes reported or subgroups analyzed prespecified (i.e., identified before analyses were conducted)? D14: Were all randomized participants analyzed in the group to which they were originally assigned (i.e., did they use an intention-to-treat analysis)? (**b**) D1: Was the research question or objective in this paper clearly stated? D2: Was the study population clearly specified and defined? D3: Was the participation rate of eligible persons at least 50%? D4: Were all the subjects selected or recruited from the same or similar populations (including the same time period)? Were inclusion and exclusion criteria for being in the study prespecified and applied uniformly to all participants? D5: Was a sample size justification, power description, or variance and effect estimates provided? D6: For the analyses in this paper, were the exposure(s) of interest measured prior to the outcome(s) being measured? D7: Was the timeframe sufficient, such that one could reasonably expect to see an association between exposure and outcome if it existed? D8: For exposures that can vary in amount or level, did the study examine different levels of the exposure as related to the outcome (e.g., categories of low exposure or exposure measured as a continuous variable)? D9: Were the exposure measures (independent variables) clearly defined, valid, reliable, and implemented consistently across all study participants? D10: Was the exposure(s) assessed more than once over time? D11: Were the outcome measures (dependent variables) clearly defined, valid, reliable, and implemented consistently across all study participants? D12: Were the outcome assessors blinded to the exposure status of participants? D13: Was the loss to follow-up after baseline 20% or less? D14: Were key potential confounding variables measured and adjusted statistically for their impact on the relationship between exposure(s) and outcome(s)?

Of the included studies, 21 were identified as having a “Low risk of bias” [38,39,40,41,42,43,44,45,46,47,48,49,50,51,52,53,54,55,56,57,58], while eight were classified as having a “Moderate risk of bias” [59,60,61,62,63,64,65,66]. Results were displayed graphically using the Traffic Light Plot (Figure 1a,b) and a Summary Plot for RCTs and cohort studies (Supplementary Materials Figure S1a,b), respectively.

### 2.4. Statistical Analysis

Two researchers undertook a synthesis of both qualitative and quantitative data. Any discrepancies or inconsistencies identified during this process were addressed through open discussion and consensus within the research team. For conducting the meta-analysis, we used the STATA MP 18.5 software (StataCorp LLC, College Station, TX, USA), known for its comprehensive statistical capabilities. Heterogeneity among studies was rigorously evaluated using the I^2^ statistic and the χ^2^ test, considering heterogeneity statistically significant when *p* < 0.1 for the χ^2^ test. We interpreted I^2^ values at 25%, 50%, and 75% as indicative of low, moderate, and high heterogeneity levels, respectively. In cases where moderate to high heterogeneity was observed, a random-effect model was applied to the meta-analysis, while a fixed-effect model was selected when heterogeneity was low. The outcomes of the study are summarized as effect measures.

In the meta-analyses, odds ratios (ORs) were regarded as statistically significant if their confidence intervals did not contain the value “1”, indicating reduced imprecision when compared to individual studies.

To examine sources of variability, stratified analyses were performed according to study quality. Sensitivity analyses were also conducted to assess the robustness of the pooled estimates by sequentially removing individual studies. Publication bias was assessed visually with a funnel plot, and if asymmetry was detected, further analyses using the trim-and-fill method were undertaken to explore and correct for bias. Moreover, Egger’s linear regression test was applied to detect potential publication bias, with a threshold *p*-value of <0.05 indicating possible bias.

## 3. Results

**Figure 2:**
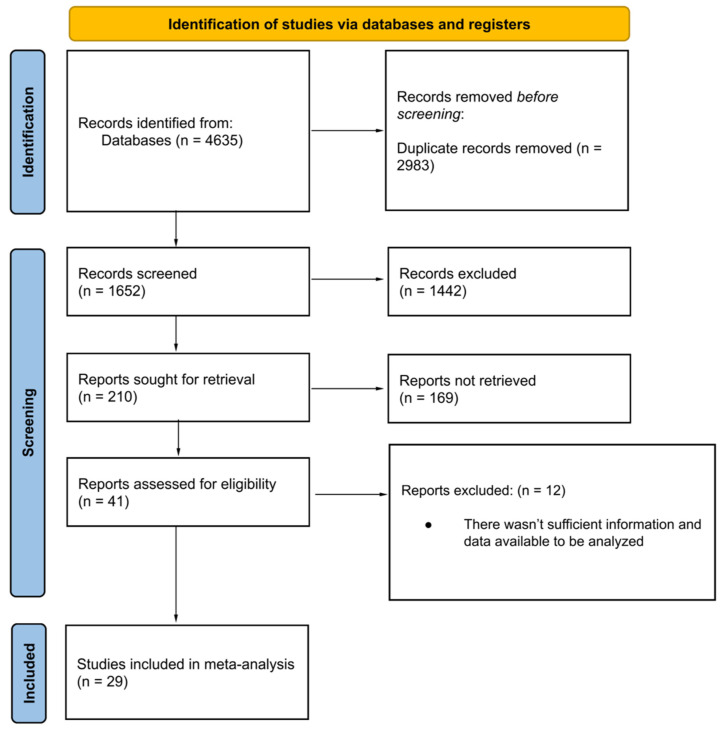
PRISMA 2020 flow diagram of study selection, inclusion, and synthesis.

A total of 29 publications were included in the analysis (Figure 2). Of these, 21 were randomized controlled trials (RCTs) [38,39,40,42,43,44,46,47,48,49,50,51,53,54,55,56,57,58,64,65,66], while the remaining studies were classified as analytical studies [41,45,52,59,60,61,62,63]. Mortality was assessed in nineteen RCTs [38,39,40,42,43,44,46,47,48,49,50,51,53,54,55,56,57,64,65] and seven analytical studies [41,45,52,60,61,62,63], ICU admissions in fourteen RCTs [40,42,43,44,46,48,49,50,53,54,55,57,65,66] and five analytical studies [41,45,52,59,61], hospital length of stay (LOS) exclusively in RCTs [42,43,44,46,48,49,50,51,53,54,56,57,58,64,65,66] and intubation rates in nine RCTs [42,43,44,47,50,53,54,57,64] and three analytical studies [60,62,63].

The main features of the studies included in the meta-analysis are reported in Supplementary Material (Supplementary Material Table S1). The main aspects, such as study design, setting, participant demographics, and details on vitamin D supplementation, are highlighted. In addition, information on the number of participants, age (reported as mean ± standard deviation or median and interquartile range (IQR)), and sex distribution (absolute numbers and percentages) are given.

More precise information on the regimens of vitamin D administered and the types of comparison used between the treatment arms of each study are reported in Supplementary material (Supplementary Material Table S2). Vitamin D dosages varied considerably, as the regimens included daily, weekly, and monthly doses. For example, in the study by Annweiler C et al. 2020 [38], 80,000 IU/day of vitamin D3 was used in the intervention group, while the control group received no vitamin D supplementation. In contrast, the study by Entrenas Castillo M et al. 2020 [40] used calcifediol with an initial dosage of 21,280 IU/day, followed by a maintenance dose of 10,640 IU/day, compared to a placebo administered in the control group.

In these studies, outcomes of all-cause mortality, intensive care unit admission, and intubation are reported, with event rates reported as percentages and absolute numbers (n/N) for both the intervention and control groups. For example, in the study by Giannini S et al. 2021 [61], in the intervention group that received 200,000 IU/day of cholecalciferol, the mortality rate was 30.56% compared to 20% in the control group that did not receive vitamin D supplementation. 

As shown in Table S1, overall mortality was assessed in most of the studies, while intensive care unit admissions and intubations were assessed less frequently. Table S2 shows that the most common dosing regimens were high doses of cholecalciferol or calcifediol, while calcitriol supplementation was the least frequent.

### 3.1. ICU Admission

The analysis of ICU admission rates provides some of the most compelling evidence for the potential benefits of vitamin D supplementation in COVID-19 patients. This outcome is particularly important as ICU admissions not only reflect the severity of the disease but also have significant implications for healthcare resource utilization and patient outcomes. Our meta-analysis examined this crucial endpoint using data from both randomized controlled trials (RCTs) and analytical studies, offering a comprehensive view of the potential impact of vitamin D supplementation on ICU admission rates.

The meta-analysis of 14 RCTs [40,42,43,44,46,48,49,50,53,54,55,57,65,66] yielded an odds ratio of 0.55 (95% CI: 0.37 to 0.79, *p* = 0.001) for ICU admission (Supplementary Material Table S3).

This result indicates a statistically significant 45% reduction in the odds of ICU admission associated with vitamin D supplementation. A high vitamin D supplementation regimen was found to be more protective than a low regimen (OR = 0.37, 95% CI: 0.20 to 0.68, *p* = 0.001). To further elucidate the nuances of this effect, we conducted several subgroup analyses.

**Figure 3:**
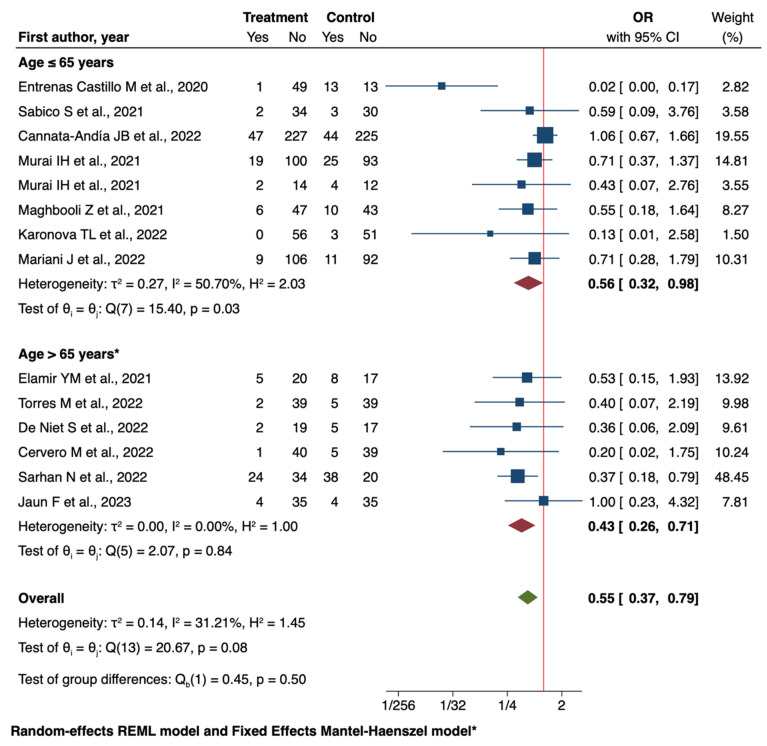
Forest plot of Impact of Vitamin D supplementation on ICU admission by age only for RCT studies. * We used a Fixed Effect Mantel-Haenszel model for age > 65 years [40,42,43,44,46,48,49,50,53,54,55,57,65,66].

When stratified by age, both younger (≤65 years) and older (>65 years) patients showed significant benefits from vitamin D supplementation. The odds ratio for patients aged 65 years or younger was 0.56 (95% CI: 0.32 to 0.98), while for those over 65, it was 0.43 (95% CI: 0.26 to 0.71). These results suggest that vitamin D supplementation may have a protective role against ICU admission across age groups, with a potentially more pronounced effect in older patients (Figure 3).

Another critical subgroup analysis was based on COVID-19 severity. Interestingly, vitamin D supplementation showed significant protective effects in non-severe cases (OR = 0.67, 95% CI: 0.51 to 0.88). This effect was not observed in cases of SARS-CoV-2 infection with severe presentation (OR = 0.22, 95% CI: 0.02 to 2.07).

**Figure 4:**
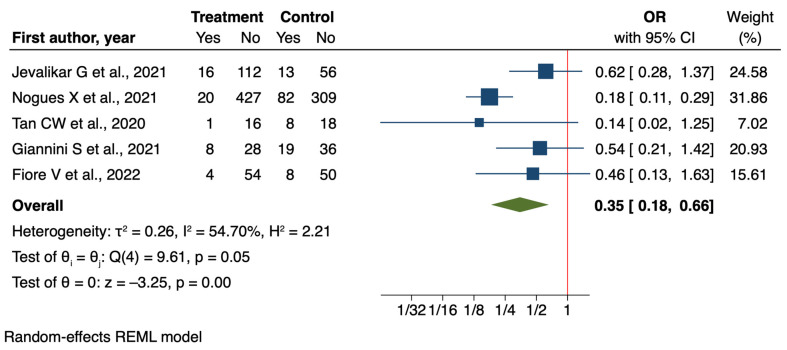
Forest plot of Impact of Vitamin D supplementation on ICU admission for analytical studies [41,45,52,59,61].

Regarding the analytical studies, we find further support for the protective effect of vitamin D supplementation against ICU admission. The meta-analysis of five analytical studies [41,45,52,59,61] showed an even more pronounced effect than the RCTs, with an odds ratio of 0.35 (95% CI: 0.18 to 0.66, *p* = 0.001). This result suggests a 65% reduction in the odds of ICU admission associated with vitamin D supplementation (Figure 4).

### 3.2. Mortality

The analysis of mortality outcomes reveals a complex picture with discrepancies between RCTs and analytical studies. The meta-analysis of nineteen RCTs [38,39,40,42,43,44,46,47,48,49,50,51,53,54,55,56,57,64,65] showed a decreased mortality with vitamin D supplementation (OR = 0.80, 95% CI: 0.61 to 1.04), but this effect did not reach statistical significance (Supplementary Material Table S3).

**Figure 5:**
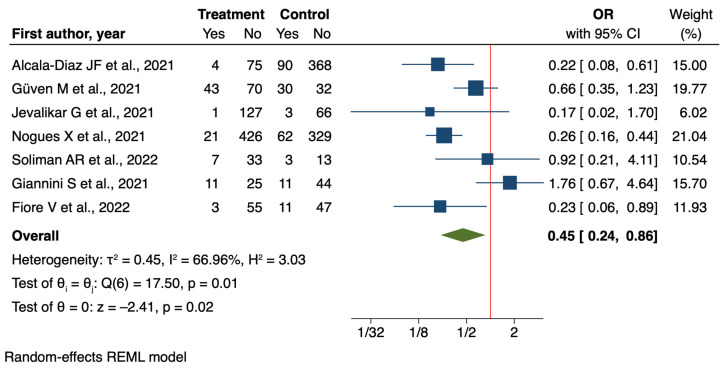
Forest plot of Impact of Vitamin D supplementation on mortality for analytical studies [41,45,52,60,61,62,63].

In contrast, the analysis of seven analytical studies [41,45,52,60,61,62,63] demonstrated a significant protective effect of vitamin D supplementation on mortality (OR = 0.45, 95% CI: 0.24 to 0.86, *p* = 0.02) (Figure 5).

This discrepancy between RCTs and analytical studies is intriguing and could be attributed to various factors. Analytical studies may be more susceptible to confounding variables and selection bias, potentially leading to an overestimation of the treatment effect. Conversely, the more rigorous design of RCTs might provide a more accurate, albeit conservative, estimate of the true effect.

Subgroup analyses of the RCTs offer additional nuance to these findings. When stratified by age, older patients (>65 years) patients showed significant reductions in mortality (OR = 0.58, 95% CI: 0.39 to 0.86). In contrast, the effect was not reported for patients younger than 65 years of age (OR = 1.05, 95% CI: 0.73 to 1.53). 

This is particularly noteworthy given that the overall RCT analysis did not reach statistical significance, suggesting that age-specific effects may be masked in the aggregate analysis.

Moreover, when vitamin D was supplemented, mortality was reduced more in the early pandemic period (February 2020–May 2020) compared to later periods.

The subgroup analysis based on COVID-19 severity revealed a marked difference in the effect of vitamin D supplementation. In patients with severe COVID-19, there was a significant reduction in mortality (OR = 0.50, 95% CI: 0.31 to 0.82). However, in non-severe cases, no benefit was observed (OR = 0.99, 95% CI: 0.71 to 1.38). This finding suggests that the potential mortality benefit of vitamin D supplementation may be most pronounced in critically ill COVID-19 patients.

The divergent results between overall RCT findings and subgroup analyses highlight the complexity of interpreting mortality data in this context. The primary analysis of the RCT suggests a potential benefit that, however, is not statistically significant, while the subgroup analyses indicate significant protective effects in specific patient populations, particularly older individuals and those with severe COVID-19.

### 3.3. Intubation

**Figure 6:**
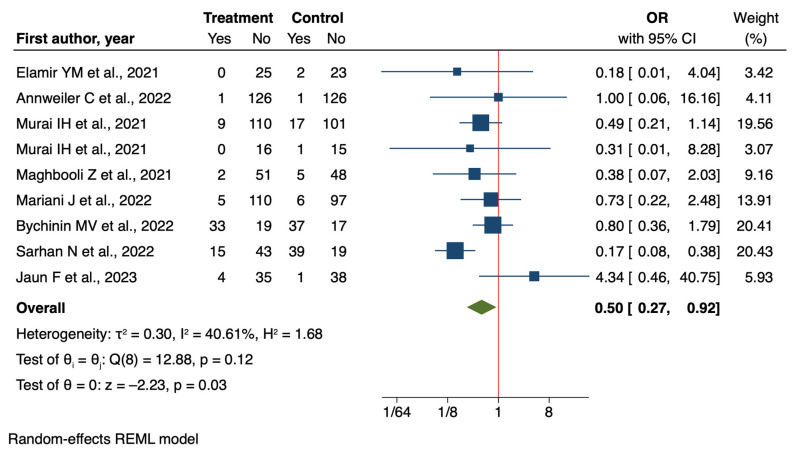
Forest plot of Impact of Vitamin D supplementation on risk of intubation for RCT studies [42,43,44,47,50,53,54,57,64].

The analysis of intubation rates provides further evidence for the potential benefits of vitamin D supplementation in COVID-19 patients. The meta-analysis of nine RCTs [42,43,44,47,50,53,54,57,64] demonstrated a significant reduction in intubation rates associated with vitamin D supplementation (OR = 0.50, 95% CI: 0.27 to 0.92, *p* = 0.03) (Figure 6).

The analysis of analytical studies, which was limited to three studies [60,62,63] focusing on non-severe COVID-19 patients aged 65 years and older, indicates a reduction in intubation rates with vitamin D supplementation (OR = 0.65, 95% CI: 0.39 to 1.08, *p* = 0.09) (Supplementary Material Table S3). However, this result did not reach statistical significance. The discrepancy in statistical significance between RCTs and analytical studies warrants careful interpretation. The analytical studies were limited to a specific subgroup of patients, which may explain the difference in results. Additionally, the smaller number of analytical studies compared to RCTs could have reduced the statistical power to detect a significant effect.

Despite these differences, both RCTs and analytical studies indicate a potential protective effect of vitamin D supplementation against the need for intubation in COVID-19 patients. This finding aligns with the results observed for ICU admission rates, suggesting that vitamin D supplementation might help reduce the need for intensive respiratory support in COVID-19 patients.

The consistency of results across different study designs, despite variations in statistical significance, strengthens the potential role of vitamin D in mitigating severe respiratory complications in COVID-19 patients. However, the observed variation suggests that the magnitude of this effect may vary depending on factors not captured in this analysis, such as baseline vitamin D status, timing of supplementation, or specific patient characteristics.

These results, while promising, underscore the need for further research to clarify the precise impact of vitamin D supplementation on intubation rates in different subgroups of COVID-19 patients. Further well-designed, stratified studies considering relevant factors could help to refine our understanding of this potential benefit and inform clinical guidelines for vitamin D use in COVID-19 management, including optimal dosing regimens and target patient population.

### 3.4. Hospital Length of Stay

The meta-analysis of 16 randomized controlled trials (RCTs) [42,43,44,46,48,49,50,51,53,54,56,57,58,64,65,66] examining the effect of vitamin D supplementation on hospital length of stay in COVID-19 patients showed a reduction in LOS, with a mean difference of −0.62 days [95% CI −1.41 to 0.18] (Supplementary Material Table S4). Subgroup analyses provided further insights into the potential moderating factors of this effect. When stratified by age, the analysis revealed a more pronounced effect in patients over 65 years (mean difference = −1.54 days, 95% CI: −1.42 to 0.18) compared to those 65 years and younger (mean difference = −0.29 days, 95% CI: −0.90 to 0.32). Although the difference between these age subgroups was not statistically significant, it suggests that older patients might derive greater benefit from vitamin D supplementation in terms of reduced hospital stay.

**Figure 7:**
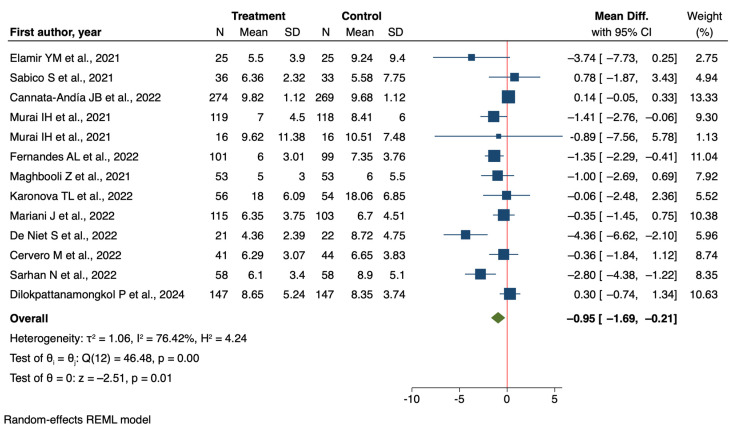
Forest plot of Impact of Vitamin D supplementation on Hospital Length of Stay [42,43,44,46,48,49,50,51,53,54,58,65,66].

Perhaps the most striking finding emerged from the subgroup analysis based on COVID-19 severity. In patients with non-severe COVID-19, vitamin D supplementation was associated with a statistically significant reduction in hospital stay (mean difference = −0.95 days, 95% CI: −1.69 to −0.21) (Figure 7).

Conversely, in severe cases, there was a non-significant increase in LOS (mean difference = 2.59 days, 95% CI: −0.90 to 6.08). The difference between these severity subgroups approached statistical significance (*p* = 0.05), highlighting the potential importance of disease severity in modulating the effect of vitamin D supplementation.

These results present a nuanced picture of the impact of vitamin D supplementation on hospital length of stay. While the overall trend suggests a potential benefit, the high heterogeneity between studies and lack of statistical significance in the primary analysis call for cautious interpretation. The subgroup analyses, particularly regarding disease severity, provide valuable insights that could guide future research and inform clinical decision-making.

## 4. Discussion

This meta-analysis provides a comprehensive evaluation of the effects of vitamin D supplementation on key clinical outcomes in COVID-19 patients. The study’s findings suggest potential benefits of vitamin D supplementation, particularly in reducing ICU admission rates and possibly intubation rates. The evidence for effects on hospital length of stay and mortality showed mixed results, with analytical studies indicating significant benefits for mortality and subgroup analyses revealing potential benefits in specific patient populations.

A crucial consideration in interpreting these results is the pharmacokinetic differences between vitamin D supplementation forms. Several analytical studies utilized calcifediol rather than cholecalciferol, which offers more rapid increases in serum 25(OH)D concentrations. This pharmacokinetic advantage may be particularly relevant in the acute setting of COVID-19, where rapid improvement in vitamin D status could be beneficial. Furthermore, emerging evidence suggests that serum 25(OH)D concentrations tend to decrease during COVID-19 infection, as demonstrated by Karonova et al. [66] in unsupplemented patients. This decline suggests active vitamin D consumption during the immune response against SARS-CoV-2, potentially supporting the rationale for supplementation during acute infection.

One of the strengths of this meta-analysis is the inclusion of both randomized controlled trials (RCTs) and analytical studies, providing a broad evidence base, consistent findings across different study designs for some outcomes, particularly ICU admission rates, detailed subgroup analyses that offer insights into the differential effects of vitamin D supplementation based on factors such as age and disease severity and finally a large pooled sample size, enhancing the statistical power of the analysis.

However, several limitations should be considered when interpreting these results. First, the high heterogeneity in some analyses, particularly for hospital length of stay, indicates substantial variability in effects across studies. Second is the ever-present potential publication bias, as studies with positive findings may be more likely to be published. Third, the variability in vitamin D dosing regimens, timing of supplementation, and baseline vitamin D status across studies may influence the observed effects. In particular, most studies did not report the duration of symptoms before hospital admission, a factor that could significantly influence both disease severity at presentation and baseline vitamin D status. However, this limitation is partially mitigated by our analysis of different treatment settings. Following standard clinical guidelines and local healthcare protocols, patients were typically allocated to different care settings based on disease severity—with more severe cases admitted to ICU and less severe cases to regular wards. This systematic triage process provides an indirect standardization of disease severity across studies, helping to partially control for variations in pre-admission disease progression. Nevertheless, the information gap regarding pre-admission symptom duration still limits our ability to determine optimal timing for vitamin D supplementation. Finally, the discrepancies between RCT and analytical study results for some outcomes, such as mortality, warrant careful interpretation.

Comparing our results with recent meta-analyses, we found concordance with the results reported by Kow et al. [67] and Argano et al. [68], who found a reduction in mortality associated with vitamin D supplementation. However, our analysis offers a more nuanced perspective, highlighting how the effect can vary significantly among different patient subgroups and disease severity levels. Yang et al.’s meta-analysis [69] reported similar results to ours regarding ICU admission and the need for mechanical ventilation, showing a reduced risk associated with higher vitamin D levels. However, they found a stronger association with mortality than our analysis. Bignardi et al.’s work [70] emphasized the importance of adjusting for confounding factors, a point that our analysis also highlights. When we analyzed studies with and without adjustment for confounders separately, only those without adjustment showed a significant association. This suggests that confounding factors may play a crucial role in determining this association, a nuance not captured in some other meta-analyses. Argano et al. [68] employed trial sequential analysis (TSA) to evaluate the robustness of the evidence. Their TSA concluded that the association between vitamin D supplementation and reduced risk of ICU admission is conclusive, while further studies are needed to confirm the effect on mortality. This aligns with our findings of stronger evidence for ICU admission reduction compared to mortality benefits.

In conclusion, while this meta-analysis suggests potential benefits of vitamin D supplementation in COVID-19 patients, particularly in reducing ICU admissions, the evidence varies across outcomes and patient subgroups. The differing effects based on age, disease severity, and possibly baseline vitamin D status highlight the need for a nuanced approach to vitamin D supplementation in COVID-19 management.

These findings underscore the need for further large-scale, well-designed, randomized controlled trials that account for baseline vitamin D status, use standardized supplementation protocols, and examine a comprehensive range of clinically relevant outcomes. Future studies should also explore the efficacy of different vitamin D dosing regimens and the timing of supplementation. Such research would help clarify the role of vitamin D supplementation in COVID-19 treatment and potentially inform clinical guidelines for its use in this context, particularly in specific patient subgroups.

## 5. Conclusions

This comparative analysis of RCTs and analytical studies on vitamin D supplementation in SARS-CoV-2 patients reveals a complex picture of potential benefits across various clinical outcomes. The most consistent finding across study types is a significant reduction in ICU admissions. Results for mortality and intubation rates are less conclusive, with discrepancies between RCTs and analytical studies highlighting the importance of study design. Hospital length of stay shows non-significant trends towards reduction, with subgroup analyses suggesting potential benefits in specific patient populations. These varied results underscore the need for further well-designed RCTs that account for factors such as baseline vitamin D status, supplementation regimens, and patient characteristics. While the evidence suggests potential benefits of vitamin D supplementation, particularly in reducing ICU admissions, the varying strength of evidence across outcomes necessitates a nuanced approach to its use in COVID-19 management and guideline development.

This comparative analysis of RCTs and analytical studies on vitamin D supplementation in SARS-CoV-2 patients reveals a complex picture of potential benefits across various clinical outcomes. The most consistent and robust finding across study types is a significant reduction in ICU admissions, with RCTs showing a 45% reduction (OR = 0.55, 95% CI: 0.37–0.79) and analytical studies demonstrating an even stronger effect (OR = 0.35, 95% CI: 0.18–0.66). Results for mortality and intubation rates present a more nuanced picture, with notable discrepancies between RCTs and analytical studies. This disparity may be attributed to differences in study design, patient populations, and timing of intervention. Importantly, subgroup analyses revealed that older patients (>65 years) and those with severe COVID-19 showed more pronounced benefits, suggesting that targeted supplementation strategies may be more effective than a one-size-fits-all approach. Hospital length of stay showed non-significant trends toward reduction in the overall analysis but demonstrated significant benefits in specific subgroups, particularly in patients with non-severe COVID-19 (mean difference = −0.95 days, 95% CI: −1.69 to −0.21). This finding suggests that early intervention with vitamin D supplementation may be more effective than later treatment in severe cases.

In clinical practice, these findings suggest that vitamin D supplementation may be most beneficial when implemented early in the disease course, particularly in high-risk populations such as older adults and those with vitamin D deficiency. However, supplementation protocols should be tailored to individual patient characteristics and disease severity, with careful consideration of timing and dosage.

## Figures and Tables

**Table 1:** Search strategy adopted in the present systematic review and meta-analysis.

Search Strategy	Details	
Search string	(“COVID-19” OR “SARS-CoV-2” OR “coronavirus” OR “2019-nCoV”) AND (“vitamin D” OR “cholecalciferol” OR “calcitriol”)	
Inclusion criteria	P (patients/population):	Patients with COVID-19 diagnosis
	I (intervention/exposure):	Patients with COVID-19 infection supplemented with Vitamin D
	C (comparisons/comparators):	Patients diagnosed with COVID-19 who received standard therapy, lower dose, no therapy or placebo
	O (outcome):	Mortality, ICU admission, intubation, and hospital length of stay
	S (study design)	RCT, cohort, and quasi-experimental studies were considered
Databases	PubMed/MEDLINE, Scopus, Cochrane, and Google Scholar	
Exclusion criteria	Items not directly pertinent to the query string; studies did not have sufficient information and data available to be analyzed; articles not meeting the PICOS criteria	
Time filter	None (from inception)	
Language filter	None (any language)	

## Data Availability

The data presented in this study are available on request from the corresponding author due to internal regulations.

## Supplementary Materials

The following supporting information can be downloaded at https://www.mdpi.com/article/10.3390/nu16223794/s1. File S1: PRISMA Checklist (Reference [71] is cited in the supplementary materials). Figure S1: Summary plot of the risk of bias for analytical studies (a) and RCT Studies (b). Table S1: Characteristics of studies included in the meta-analysis. Table S2: Characteristics of the study outcomes included in the meta-analysis. Table S3: Risk ratio (RR) and 95% CI for all meta-analyses carried out (mortality, ICU admission, and intubation). Table S4: Mean difference and 95% CI for all meta-analyses carried out (LOS).

## Abbreviations

ICU	intensive care unit
IC	confidence interval
LOS	hospital length of stay
OR	Odds Ratio
RCT	randomized control trial

## References

[1] Molina P., Carrero J.J., Bover J., Chauveau P., Mazzaferro S., Torres P.U., European Renal Nutrition (ERN) and Chronic Kidney Disease-Mineral and Bone Disorder (CKD-MBD) Working Groups of the European Renal Association-European Dialysis Transplant Association (ERA-EDTA) (2017). Vitamin D, a modulator of musculoskeletal health in chronic kidney disease. J. Cachexia Sarcopenia Muscle.

[2] Umar M., Sastry K.S., Chouchane A.I. (2018). Role of Vitamin D Beyond the Skeletal Function: A Review of the Molecular and Clinical Studies. Int. J. Mol. Sci..

[3] Bover J., Ruiz C.E., Pilz S., Dasilva I., Díaz M.M., Guillén E., Ureña Torres P., Cozzolino M., Vervloet M. (2016). Vitamin D Receptor and Interaction with DNA: From Physiology to Chronic Kidney Disease. Vitamin D in Chronic Kidney Disease.

[4] Haussler M.R., Haussler C.A., Bartik L., Whitfield G.K., Hsieh J.C., Slater S., Jurutka P.W. (2008). Vitamin D receptor: Molecular signaling and actions of nutritional ligands in disease prevention. Nutr. Rev..

[5] Sirajudeen S., Shah I., Al Menhali A. (2019). A Narrative Role of Vitamin D and Its Receptor: With Current Evidence on the Gastric Tissues. Int. J. Mol. Sci..

[6] Shirvani A., Kalajian T.A., Song A., Holick M.F. (2019). Disassociation of Vitamin D’s Calcemic Activity and Non-calcemic Genomic Activity and Individual Responsiveness: A Randomized Controlled Double-Blind Clinical Trial. Sci. Rep..

[7] Voltan G., Cannito M., Ferrarese M., Ceccato F., Camozzi V. (2023). Vitamin D: An Overview of Gene Regulation, Ranging from Metabolism to Genomic Effects. Genes.

[8] van de Peppel J., van Leeuwen J.P. (2014). Vitamin D and gene networks in human osteoblasts. Front. Physiol..

[9] Bouillon R., Marcocci C., Carmeliet G., Bikle D., White J.H., Dawson-Hughes B., Lips P., Munns C.F., Lazaretti-Castro M., Giustina A. (2019). Skeletal and Extraskeletal Actions of Vitamin D: Current Evidence and Outstanding Questions. Endocr. Rev..

[10] Christakos S., DeLuca H.F. (2011). Minireview: Vitamin D: Is there a role in extraskeletal health?. Endocrinology.

[11] Girgis C.M., Clifton-Bligh R.J., Hamrick M.W., Holick M.F., Gunton J.E. (2013). The roles of vitamin D in skeletal muscle: Form, function, and metabolism. Endocr. Rev..

[12] Dzik K.P., Kaczor J.J. (2019). Mechanisms of vitamin D on skeletal muscle function: Oxidative stress, energy metabolism and anabolic state. Eur. J. Appl. Physiol..

[13] Muñoz A., Grant W.B. (2022). Vitamin D and Cancer: An Historical Overview of the Epidemiology and Mechanisms. Nutrients.

[14] Zhang H., Zhang H., Wen X., Zhang Y., Wei X., Liu T. (2015). Vitamin D Deficiency and Increased Risk of Bladder Carcinoma: A Meta-Analysis. Cell. Physiol. Biochem..

[15] Dawson-Hughes B., Staten M.A., Knowler W.C., Nelson J., Vickery E.M., LeBlanc E.S., Neff L.M., Park J., Pittas A.G., D2D Research Group (2020). Intratrial Exposure to Vitamin D and New-Onset Diabetes Among Adults with Prediabetes: A Secondary Analysis From the Vitamin D and Type 2 Diabetes (D2d) Study. Diabetes Care.

[16] Aiello G., Lombardo M., Baldelli S. (2024). Exploring Vitamin D Synthesis and Function in Cardiovascular Health: A Narrative Review. Appl. Sci..

[17] Gaudet M., Plesa M., Mogas A., Jalaleddine N., Hamid Q., Al Heialy S. (2022). Recent advances in vitamin D implications in chronic respiratory diseases. Respir. Res..

[18] Port Louis L.R., Kannan S., Shanmugham D., Balakrishnan J., Nagarajan P., Tappia P.S., Shah A.K., Dhalla N.S. (2024). Vitamin D and Immune Function: Unraveling the Connections. Lipophilic Vitamins in Health and Disease. Advances in Biochemistry in Health and Disease.

[19] Papagni R., Pellegrino C., Di Gennaro F., Patti G., Ricciardi A., Novara R., Cotugno S., Musso M., Guido G., Ronga L. (2022). Impact of Vitamin D in Prophylaxis and Treatment in Tuberculosis Patients. Int. J. Mol. Sci..

[20] Delvin E., Souberbielle J.C., Viard J.P., Salle B. (2014). Role of vitamin D in acquired immune and autoimmune diseases. Crit. Rev. Clin. Lab. Sci..

[21] Wu H.X., Xiong X.F., Zhu M., Wei J., Zhuo K.Q., Cheng D.Y. (2018). Effects of vitamin D supplementation on the outcomes of patients with pulmonary tuberculosis: A systematic review and meta-analysis. BMC Pulm. Med..

[22] Jolliffe D.A., Ganmaa D., Wejse C., Raqib R., Haq M.A., Salahuddin N., Daley P.K., Ralph A.P., Ziegler T.R., Martineau A.R. (2019). Adjunctive vitamin D in tuberculosis treatment: Meta-analysis of individual participant data. Eur. Respir. J..

[23] Reuter A., Furin J. (2020). The problem with vitamin D supplementation for tuberculosis. Lancet HIV.

[24] Martineau A.R., Jolliffe D.A., Hooper R.L., Greenberg L., Aloia J.F., Bergman P., Dubnov-Raz G., Esposito S., Ganmaa D., Ginde A.A. (2017). Vitamin D supplementation to prevent acute respiratory tract infections: Systematic review and meta-analysis of individual participant data. BMJ.

[25] Gallagher J.C. (2021). Vitamin D and respiratory infections. Lancet Diabetes Endocrinol..

[26] Jolliffe D.A., Greenberg L., Hooper R.L., Mathyssen C., Rafiq R., de Jongh R.T., Camargo C.A., Griffiths C.J., Janssens W., Martineau A.R. (2019). Vitamin D to prevent exacerbations of COPD: Systematic review and meta-analysis of individual participant data from randomised controlled trials. Thorax.

[27] Vassalle C. (2024). Editorial: Vitamin D: From pathophysiology to clinical impact. Front. Nutr..

[28] Chiodini I., Gatti D., Soranna D., Merlotti D., Mingiano C., Fassio A., Adami G., Falchetti A., Eller-Vainicher C., Rossini M. (2021). Vitamin D Status and SARS-CoV-2 Infection and COVID-19 Clinical Outcomes. Front. Public Health.

[29] Cutolo M., Paolino S., Smith V. (2020). Evidences for a protective role of vitamin D in COVID-19. RMD Open.

[30] Sartini M., Del Puente F., Oliva M., Carbone A., Bobbio N., Schinca E., Giribone L., Cristina M.L. (2024). Preventive Vitamin D Supplementation and Risk for COVID-19 Infection: A Systematic Review and Meta-Analysis. Nutrients.

[31] Sartini M., Del Puente F., Oliva M., Carbone A., Blasi Vacca E., Parisini A., Boni S., Bobbio N., Feasi M., Battistella A. (2021). Riding the COVID Waves: Clinical Trends, Outcomes, and Remaining Pitfalls of the SARS-CoV-2 Pandemic: An Analysis of Two High-Incidence Periods at a Hospital in Northern Italy. J. Clin. Med..

[32] Nicolae M., Mihai C.M., Chisnoiu T., Balasa A.L., Frecus C.E., Mihai L., Lupu V.V., Ion I., Pantazi A.C., Nelson Twakor A. (2023). Immunomodulatory Effects of Vitamin D in Respiratory Tract Infections and COVID-19 in Children. Nutrients.

[33] Page M.J., Moher D., Bossuyt P.M., Boutron I., Hoffmann T.C., Mulrow C.D., Shamseer L., Tetzlaff J.M., Akl E.A., Brennan S.E. (2021). PRISMA 2020 explanation and elaboration: Updated guidance and exemplars for reporting systematic reviews. BMJ.

[34] Luo D., Wan X., Liu J., Tong T. (2018). Optimally estimating the sample mean from the sample size, median, mid-range, and/or mid-quartile range. Stat. Methods Med. Res..

[35] Wan X., Wang W., Liu J., Tong T. (2014). Estimating the sample mean and standard deviation from the sample size, median, range and/or interquartile range. BMC Med. Res. Methodol..

[36] National Heart, Lung and Blood Institute Study Quality Assessment Tools. https://www.nhlbi.nih.gov/health-topics/study-quality-assessment-tools.

[37] JBI’s Critical Appraisal Tools Assist in Assessing the Trustworthiness, Relevance and Results of Published Papers. JBI Critical Appraisal Tool for Quasi-Experimental Studies. https://jbi.global/critical-appraisal-tools.

[38] Annweiler C., Hanotte B., Grandin de l’Eprevier C., Sabatier J.M., Lafaie L., Célarier T. (2020). Vitamin D and survival in COVID-19 patients: A quasi-experimental study. J. Steroid Biochem. Mol. Biol..

[39] Annweiler G., Corvaisier M., Gautier J., Dubée V., Legrand E., Sacco G., Annweiler C. (2020). Vitamin D Supplementation Associated to Better Survival in Hospitalized Frail Elderly COVID-19 Patients: The GERIA-COVID Quasi-Experimental Study. Nutrients.

[40] Entrenas Castillo M., Entrenas Costa L.M., Vaquero Barrios J.M., Alcalá Díaz J.F., López Miranda J., Bouillon R., Quesada Gomez J.M. (2020). Effect of calcifediol treatment and best available therapy versus best available therapy on intensive care unit admission and mortality among patients hospitalized for COVID-19: A pilot randomized clinical study. J. Steroid Biochem. Mol. Biol..

[41] Jevalikar G., Mithal A., Singh A., Sharma R., Farooqui K.J., Mahendru S., Dewan A., Budhiraja S. (2021). Lack of association of baseline 25-hydroxyvitamin D levels with disease severity and mortality in Indian patients hospitalized for COVID-19. Sci. Rep..

[42] Maghbooli Z., Sahraian M.A., Jamalimoghadamsiahkali S., Asadi A., Zarei A., Zendehdel A., Varzandi T., Mohammadnabi S., Alijani N., Karimi M. (2021). Treatment with 25-Hydroxyvitamin D3 (Calcifediol) Is Associated with a Reduction in the Blood Neutrophil-to-Lymphocyte Ratio Marker of Disease Severity in Hospitalized Patients With COVID-19: A Pilot Multicenter, Randomized, Placebo-Controlled, Double-Blinded Clinical Trial. Endocr. Pract..

[43] Murai I.H., Fernandes A.L., Sales L.P., Pinto A.J., Goessler K.F., Duran C.S.C., Silva C.B.R., Franco A.S., Macedo M.B., Dalmolin H.H.H. (2021). Effect of a Single High Dose of Vitamin D3 on Hospital Length of Stay in Patients With Moderate to Severe COVID-19: A Randomized Clinical Trial. JAMA.

[44] Murai I.H., Fernandes A.L., Antonangelo L., Gualano B., Pereira R.M.R. (2021). Effect of a Single High-Dose Vitamin D3 on the Length of Hospital Stay of Severely 25-Hydroxyvitamin D-Deficient Patients with COVID-19. Clinics.

[45] Nogues X., Ovejero D., Pineda-Moncusí M., Bouillon R., Arenas D., Pascual J., Ribes A., Guerri-Fernandez R., Villar-Garcia J., Rial A. (2021). Calcifediol Treatment and COVID-19-Related Outcomes. J. Clin. Endocrinol. Metab..

[46] Sabico S., Enani M.A., Sheshah E., Aljohani N.J., Aldisi D.A., Alotaibi N.H., Alshingetti N., Alomar S.Y., Alnaami A.M., Amer O.E. (2021). Effects of a 2-Week 5000 IU versus 1000 IU Vitamin D3 Supplementation on Recovery of Symptoms in Patients with Mild to Moderate Covid-19: A Randomized Clinical Trial. Nutrients.

[47] Annweiler C., Beaudenon M., Gautier J., Gonsard J., Boucher S., Chapelet G., Darsonval A., Fougère B., Guérin O., Houvet M. (2022). High-dose versus standard-dose vitamin D supplementation in older adults with COVID-19 (COVIT-TRIAL): A multicenter, open-label, randomized controlled superiority trial. PLoS Med..

[48] Cervero M., López-Wolf D., Casado G., Novella-Mena M., Ryan-Murua P., Taboada-Martínez M.L., Rodríguez-Mora S., Vigón L., Coiras M., Torres M. (2022). Beneficial Effect of Short-Term Supplementation of High Dose of Vitamin D3 in Hospitalized Patients With COVID-19: A Multicenter, Single-Blinded, Prospective Randomized Pilot Clinical Trial. Front. Pharmacol..

[49] De Niet S., Trémège M., Coffiner M., Rousseau A.F., Calmes D., Frix A.N., Gester F., Delvaux M., Dive A.F., Guglielmi E. (2022). Positive Effects of Vitamin D Supplementation in Patients Hospitalized for COVID-19: A Randomized, Double-Blind, Placebo-Controlled Trial. Nutrients.

[50] Elamir Y.M., Amir H., Lim S., Rana Y.P., Lopez C.G., Feliciano N.V., Omar A., Grist W.P., Via M.A. (2022). A randomized pilot study using calcitriol in hospitalized COVID-19 patients. Bone.

[51] Fernandes A.L., Murai I.H., Reis B.Z., Sales L.P., Santos M.D., Pinto A.J., Goessler K.F., Duran C.S.C., Silva C.B.R., Franco A.S. (2022). Effect of a single high dose of vitamin D3 on cytokines, chemokines, and growth factor in patients with moderate to severe COVID-19. Am. J. Clin. Nutr..

[52] Fiore V., De Vito A., Bagella P., Princic E., Mariani A.A., Denti L., Fois A.G., Madeddu G., Babudieri S., Maida I. (2022). Effectiveness of Vitamin D Supplements among Patients Hospitalized for COVID-19: Results from a Monocentric Matched-Cohort Study. Healthcare.

[53] Mariani J., Antonietti L., Tajer C., Ferder L., Inserra F., Sanchez Cunto M., Brosio D., Ross F., Zylberman M., López D.E. (2022). High-dose vitamin D versus placebo to prevent complications in COVID-19 patients: Multicentre randomized controlled clinical trial. PLoS ONE.

[54] Sarhan N., Abou Warda A.E., Sarhan R.M., Boshra M.S., Mostafa-Hedeab G., ALruwaili B.F., Ibrahim H.S.G., Schaalan M.F., Fathy S. (2022). Evidence for the Efficacy of a High Dose of Vitamin D on the Hyperinflammation State in Moderate-to-Severe COVID-19 Patients: A Randomized Clinical Trial. Medicina.

[55] Torres M., Casado G., Vigón L., Rodríguez-Mora S., Mateos E., Ramos-Martín F., López-Wolf D., Sanz-Moreno J., Ryan-Murua P., Taboada-Martínez M.L. (2022). Changes in the immune response against SARS-CoV-2 in individuals with severe COVID-19 treated with high dose of vitamin D. Biomed. Pharmacother..

[56] Domazet Bugarin J., Dosenovic S., Ilic D., Delic N., Saric I., Ugrina I., Stojanovic Stipic S., Duplancic B., Saric L. (2023). Vitamin D Supplementation and Clinical Outcomes in Severe COVID-19 Patients-Randomized Controlled Trial. Nutrients.

[57] Jaun F., Boesing M., Luethi-Corridori G., Abig K., Bloch N., Giezendanner S., Grillmayr V., Haas P., Leuppi-Taegtmeyer A.B., Muser J. (2023). Effect of Single High Dose Vitamin D Substitution in Hospitalized COVID-19 Patients with Vitamin D Deficiency on Length of Hospital Stay. Biomedicines.

[58] Dilokpattanamongkol P., Yan C., Jayanama K., Ngamjanyaporn P., Sungkanuparph S., Rotjanapan P. (2024). Impact of vitamin D supplementation on the clinical outcomes of COVID-19 pneumonia patients: A single-center randomized controlled trial. BMC Complement. Med. Ther..

[59] Tan C.W., Ho L.P., Kalimuddin S., Cherng B.P.Z., Teh Y.E., Thien S.Y., Wong H.M., Tern P.J.W., Chandran M., Chay J.W.M. (2020). Cohort study to evaluate the effect of vitamin D, magnesium, and vitamin B12 in combination on progression to severe outcomes in older patients with coronavirus (COVID-19). Nutrition.

[60] Alcala-Diaz J.F., Limia-Perez L., Gomez-Huelgas R., Martin-Escalante M.D., Cortes-Rodriguez B., Zambrana-Garcia J.L., Entrenas-Castillo M., Perez-Caballero A.I., López-Carmona M.D., Garcia-Alegria J. (2021). Calcifediol Treatment and Hospital Mortality Due to COVID-19: A Cohort Study. Nutrients.

[61] Giannini S., Passeri G., Tripepi G., Sella S., Fusaro M., Arcidiacono G., Torres M.O., Michielin A., Prandini T., Baffa V. (2021). Effectiveness of In-Hospital Cholecalciferol Use on Clinical Outcomes in Comorbid COVID-19 Patients: A Hypothesis-Generating Study. Nutrients.

[62] Güven M., Gültekin H. (2021). The effect of high-dose parenteral vitamin D3 on COVID-19-related inhospital mortality in critical COVID-19 patients during intensive care unit admission: An observational cohort study. Eur. J. Clin. Nutr..

[63] Soliman A.R., Abdelaziz T.S., Fathy A. (2021). Impact of Vitamin D Therapy on the Progress COVID-19: Six Weeks Follow-Up Study of Vitamin D Deficient Elderly Diabetes Patients. Proc. Singap. Healthc..

[64] Bychinin M.V., Klypa T.V., Mandel I.A., Yusubalieva G.M., Baklaushev V.P., Kolyshkina N.A., Troitsky A.V. (2022). Effect of vitamin D3 supplementation on cellular immunity and inflammatory markers in COVID-19 patients admitted to the ICU. Sci. Rep..

[65] Cannata-Andía J.B., Díaz-Sottolano A., Fernández P., Palomo-Antequera C., Herrero-Puente P., Mouzo R., Carrillo-López N., Panizo S., Ibañez G.H., Cusumano C.A. (2022). A single-oral bolus of 100,000 IU of cholecalciferol at hospital admission did not improve outcomes in the COVID-19 disease: The COVID-VIT-D-a randomised multicentre international clinical trial. BMC Med..

[66] Karonova T.L., Golovatyuk K.A., Kudryavtsev I.V., Chernikova A.T., Mikhaylova A.A., Aquino A.D., Lagutina D.I., Zaikova E.K., Kalinina O.V., Golovkin A.S. (2022). Effect of Cholecalciferol Supplementation on the Clinical Features and Inflammatory Markers in Hospitalized COVID-19 Patients: A Randomized, Open-Label, Single-Center Study. Nutrients.

[67] Kow C.S., Ramachandram D.S., Hasan S.S., Wong Z., Thiruchelvam K. (2024). The impact of vitamin D administration on mortality in COVID-19 patients: A systematic review and meta-analysis of randomized controlled trials. Inflammopharmacology.

[68] Argano C., Mallaci Bocchio R., Natoli G., Scibetta S., Lo Monaco M., Corrao S. (2023). Protective Effect of Vitamin D Supplementation on COVID-19-Related Intensive Care Hospitalization and Mortality: Definitive Evidence from Meta-Analysis and Trial Sequential Analysis. Pharmaceuticals.

[69] Yang Y., Sun W., Yang F., Zhang G., Li X., Sun S., Xing Y. (2024). Therapeutic effects of vitamin D supplementation on COVID-19 aggravation: A systematic review and meta-analysis of randomized controlled trials. Front. Pharmacol..

[70] Bignardi P.R., de Andrade Castello P., de Matos Aquino B., Delfino V.D.A. (2023). Is the vitamin D status of patients with COVID-19 associated with reduced mortality? A systematic review and meta-analysis. Arch. Endocrinol. Metab..

[71] Page M.J., McKenzie J.E., Bossuyt P.M., Boutron I., Hoffmann T.C., Mulrow C.D., Shamseer L., Tetzlaff J.M., Akl E.A., Brennan S.E. (2021). The PRISMA 2020 statement: An updated guideline for reporting systematic reviews. BMJ.

