# Supplementary material for: The Effect of Vitamin D Supplementation Post COVID-19 Infection and Related Outcomes: A Systematic Review and Meta-Analysis
Source: Nutrients. 2024 Nov 5;16(22):3794. doi: 10.3390/nu16223794 (PMC11597733; doi:10.3390/nu16223794)
Supplement: Supplementary file 1 [file nutrients-16-03794-s001.zip › SUPPLEMENTARY TABLE S1-S2.pdf]

Table S1. Characteristics of studies included in meta-analysis.

References	Study Design, Setting	Partecipants	Vitamin D Supplementation Group			Control Group			Outcomes (relevant for this meta-analysis)
			No.	Age	Sex, male	No.	Age	Sex, male	
Annweiler C et al., 2020	Retrospective Quasi-Experimental; France	Elderly Patients	57	87.7 ± 9.3	12 (21.1)	9	87.4 ± 7.2	3 (32.3)	Mortality, Intubation
Annweiler G et al., 2020	Retrospective Quasi-Experimental; France	Elderly Patients	16	85 (84–89)	11 (68.7)	32	88 (84–92)	19 (59.4)	Mortality
Entrenas Castillo M et al., 2020	RCT, Spain	Patients	50	53.14 ± 10.77	27 (54.0)	26	52.77 ± 9.35	18 (69.0)	Mortality, ICU admission
Tan CW et al., 2020	Retrospective Cohort, Singapore	Elderly Patients	17	58.4 ± 7	64.7	26	64.1 ± 7.9	15 (57.7)	Mortality, ICU admission
Alcala-Diaz JF et al., 2021	Retrospective Cohort, Spain	Patients	79	69 ± 15	42 (53.0)	458	67 ± 16	275 (60.0)	Mortality, Intubation
Elamir YM et al., 2021	RCT, USA	Patients	25	69 ± 18	12 (48.0)	25	64 ± 16	13 (52.0)	Mortality, ICU admission, Intubation, LOS
Giannini S et al., 2021	Retrospective Cohort, Italy	Patients	36	73 ± 13	19 (53.0)	55	74 ± 13	31 (56.0)	Mortality, ICU admission
Güven M et al., 2021	Observational Cohort, Turkey	ICU patients	113	74 (60–81)	69 (61.1)	62	75 (62–83)	36 (58.0)	Mortality, Intubation
Jevalikar G et al., 2021	Prospective Cohort, India	Patients	128	45.5 ± 18.2	-	69	48.8 ± 14.7	-	Mortality, ICU admission, Intubation
Maghbooli Z et al., 2021	RCT, Tehran	Patients	53	50 ± 15	31 (59.0)	53	49 ± 13	33 (62.0)	Mortality, ICU admission, LOS
Murai IH et al., 2021	RCT, Brazil	Patients	119	56.5 ± 13.8	70 (58.8)	118	56.0 ± 15.0	63 (53.4)	Mortality, ICU admission, Intubation, LOS
	RCT, Brazil	Patients	16	55.7 ± 16.6	9 (56.3)	16	61.3 ± 14.4	6 (37.5)	Mortality, ICU admission, Intubation, LOS
Nogues X et al., 2021	Prospective Cohort, Spain	Patients	447	61.81 ± 15.5	264 (59.1)	391	62.41 ± 17.2	231 (59.1)	Mortality, ICU admission, Intubation

Sabico S et al., 2021	RCT, Saudi Arabia	Patients	36	46.3 ± 15.2	21 (58.3)	33	53.5 ± 12.3	13 (39.4)	Mortality, ICU admission, LOS
Annweiler C et al., 2022	RCT, France	Elderly Patients	127	87 (81 - 92)	61 (48.0)	127	89 (83 - 93)	44 (35.0)	Mortality, Intubation
Bychinin MV et al., 2022	RCT, Russia	ICU patients	52	64.5 (57–71)	22 (42.3)	54	63.5 (54–81)	31 (57.0)	Mortality, Intubation, LOS
Cannata-Andía JB et al., 2022	RCT, Spain	Patients	274	59.0 (49.0-70.0)	181 (66.1)	269	57.0 (45.0-67.0)	172 (63.9)	Mortality, ICU admission, LOS
Cervero M et al., 2022	RCT, Spain	Patients	41	64 (44–72)	30 (73.2)	44	67 (58–75)	30 (68.2)	Mortality, ICU admission, LOS
De Niet S et al., 2022	RCT, Belgium	Patients	21	63.24 ± 14.46	13 (62.0)	22	68.73 ± 10.97	10 (45.0)	Mortality, ICU admission, LOS
Fernandes AL et al., 2022	RCT, Brazil	Patients	101	55.3 ± 14.2	58 (57.4)	99	55.7 ± 14.5	51 (51.5)	Mortality, LOS
Fiore V et al., 2022	Retrospective Cohort, Italy	Patients	58	62.5 ± 14.8	33 (56.9)	58	62.9 ± 12.8	33 (56.9)	Mortality, ICU admission
Karonova TL et al., 2022	RCT, Russia	Patients	56	58 (50-65)	-	54	64 (55-70)	-	Mortality, ICU admission, LOS
Mariani J et al., 2022	RCT, Argentina	Patients	115	59.8 ± 10.7	64 (55.7)	103	58.3 ± 10.6	51 (49.5)	Mortality, ICU admission, Intubation, LOS
Sarhan N et al., 2022	RCT, Egypt	Patients	58	66.1 ± 11.2	38 (65.5)	58	65.7 ± 12.6	46 (79.3)	Mortality, ICU admission, LOS
Soliman AR et al., 2022	Prospective Cohort, Egypt	Elderly Patients	40	71.3 ± 4.16	-	16	70.19 ± 4.57	-	Mortality, Intubation
Torres M et al., 2022	RCT, Spain	Patients	41	67.0 (58.0–75.0)	30 (73.2)	44	65.3 (44.0–72.3)	30 (68.2)	Mortality, ICU admission
Domazet Bugarin J et al., 2023	RCT, Croatia	ICU patients	75	65 (59-71)	52 (69.3)	77	65.5 (39-82)	58 (75.0)	Mortality, LOS
Jaun F et al., 2023	RCT, Switzerland	Patients	39	60.49 ± 13.84	25 (64.1)	39	61.38 ± 15.29	32 (81.1)	Mortality, ICU admission, Intubation, LOS
Dilokpattanamongkol P et al., 2024	RCT, Thailand	Patients	147	47.90 ± 16.77	62 (42.2)	147	53.71 ± 18.80	75 (51.0)	LOS

NA = not applicable, LOS = Hospital Length of stay

Table S2. Characteristics of the study outcomes included in the meta-analysis.

References	Treatments Arms	All-Cause Mortality (n/N, %)		ICU Admission (n/N, %)		Intubation (n/N, %)	
		Intervention	Control	Intervention	Control	Intervention	Control
Annweiler C et al., 2020	Intervention: 80,000 IU/day of vitamin D3;	10/57	5/9	NA	NA	NA	NA
	control: no vitamin D supplementation	17.54	55.56				
Annweiler G et al., 2020	Intervention: 80,000 IU/day of vitamin D3;	3/16	10/32	NA	NA	NA	NA
	control: no vitamin D supplementation	18.75	31.25				
Entrenas Castillo M et al., 2020	Intervention: 21,280 IU/day 1, then 10,640 UI/day of calcifediol;	0/50	2/26	1/50	13/26	NA	NA
	control: placebo	0.00	7.69	2.00	50.00		
Tan CW et al., 2020	Intervention: 1000 IU/day of vitamin D3;	NA	NA	1/17	8/26	NA	NA
	control: no vitamin D supplementation			5.88	30.77		
Alcala-Diaz JF et al., 2021	Intervention: 21,280 IU/day 1, then 10,640 UI/day of calcifediol;	4/79	90/458	NA	NA	3/79	26/458
	control: no vitamin D supplementation	5.06	19.65			3.80	5.68
Elamir YM et al., 2021	Intervention: 0.5 µg/day of calcitriol;	0/25	3/25	5/25	8/25	0/25	2/25
	control: no vitamin D supplementation	0.00	12.00	20.00	32.00	0.00	8.00
Giannini S et al., 2021	Intervention: 200,000 IU/day of cholecalciferol;	11/36	11/55	8/36	19/55	NA	NA
	control: no vitamin D supplementation	30.56	20.00	22.22	34.55		
Güven M et al., 2021	Intervention: 300,000 IU of vitamin D3;	43/113	30/62	NA	NA	44/113	31/62
	control: no vitamin D supplementation	38.05	48.39			38.94	50.00
Jevalikar G et al., 2021	Intervention: 60,000 IU of cholecalciferol;	1/128	3/69	16/128	13/69	NA	NA
	control: no vitamin D supplementation	0.78	4.35	12.50	18.84		
Maghbooli Z et al., 2021	Intervention: 3000 to 6000 IU per day of calcifediol;	3/53	5/53	6/53	10/53	2/53	5/53
	control: placebo	5.66	9.43	11.32	18.87	3.77	9.43
Murai IH et al., 2021	Intervention: 200,000 IU/day of vitamin D3;	9/119	6/118	19/119	25/118	9/119	17/118
	control: placebo	7.56	5.08	15.97	21.19	7.56	14.41
	Intervention: 200,000 IU/day of vitamin D3;	0/16	1/16	2/16	4/16	0/16	1/16
	control: placebo	0.00	6.25	12.50	25.00	0.00	6.25
Nogues X et al., 2021	Intervention: 21,280 IU/day 1, then 10,640 UI/day of calcifediol;	21/447	62/391	20/447	82/391	NA	NA
	control: no vitamin D supplementation	4.70	15.86	4.47	20.97		
Sabico S et al., 2021	Intervention: 5000 IU/day of vitamin D3;	1/36	0/33	2/36	3/33	NA	NA
	control: 1000 IU/day	2.78	0.00	5.56	9.09		

Annweiler C et al., 2022	Intervention: 400,000 IU of cholecalciferol; control: 50,000 IU	8/127 6.30	14/127 11.02	NA	NA	1/127 0.79	1/127 0.79
Bychinin MV et al., 2022	Intervention: 60,000 IU of vitamin D3; control: placebo	19/52 36.54	27/54 50.00	NA	NA	33/52 63.46	37/54 68.52
Cannata-Andía JB et al., 2022	Intervention: 100,000 IU/day of cholecalciferol; control: no vitamin D supplementation	22/274 8.03	15/269 5.58	47/274 17.15	44/269 16.36	NA	NA
Cervero M et al., 2022	Intervention: 10,000 IU/day of cholecalciferol; control: 2000 IU/day	1/41 2.44	1/44 2.27	1/41 2.44	5/44 11.36	NA	NA
De Niet S et al., 2022	Intervention: 25,000 IU of cholecalciferol; control: placebo	3/21 14.29	4/22 18.18	2/21 9.52	5/22 22.73	NA	NA
Fernandes AL et al., 2022	Intervention: 200,000 IU of vitamin D3; control: placebo	9/101 8.91	6/99 6.06	NA	NA	NA	NA
Fiore V et al., 2022	Intervention: 100,000 IU/daily of vitamin D3; control: no vitamin D supplementation	3/58 5.17	11/58 18.97	4/58 6.90	8/58 13.79	NA	NA
Karonova TL et al., 2022	Intervention: 50,000 IU of cholecalciferol; control: no vitamin D supplementation	NA	NA	0/56 0.00	3/54 5.56	NA	NA
Mariani J et al., 2022	Intervention: 500,000 IU of vitamin D3; control: placebo	5/115 4.35	2/103 1.94	9/115 7.83	11/103 10.68	5/115 4.35	6/103 5.83
Sarhan N et al., 2022	Intervention: 200,000 IU of cholecalciferol; control: 1 mcg/day of D-alfacalcidol	26/58 44.83	30/58 51.72	24/58 41.38	38/58 65.52	15/58 25.86	39/58 67.24
Soliman AR et al., 2022	Intervention: 200,000 IU of vitamin D; control: placebo	7/40 17.50	3/16 18.75	NA	NA	14/40 35.00	7/16 43.75
Torres M et al., 2022	Intervention: 10,000 IU/day of cholecalciferol; control: 2000 IU/day	1/41 2.44	1/44 2.27	2/41 4.88	5/44 11.36	NA	NA
Domazet Bugarin J et al., 2023	Intervention: 10,000 IU/day of cholecalciferol; control: placebo	9/75 12.00	13/77 16.88	NA	NA	NA	NA
Jaun F et al., 2023	Intervention: 140,000 IU of vitamin D3; control: placebo	1/39 2.56	2/39 5.13	4/39 10.26	4/39 10.26	4/39 10.26	1/39 2.56
Dilokpattanamongkol P et al., 2024	Intervention: 2 mcg daily of alfacalcidol; control: placebo	NA	NA	NA	NA	NA	NA

NA = not applicable.
