# Supplementary material for: The Effect of Vitamin D Supplementation Post COVID-19 Infection and Related Outcomes: A Systematic Review and Meta-Analysis
Source: Nutrients. 2024 Nov 5;16(22):3794. doi: 10.3390/nu16223794 (PMC11597733; doi:10.3390/nu16223794)
Supplement: Supplementary file 1 [file nutrients-16-03794-s001.zip › Supplementary Table S3-S4.pdf]

**Table S3.** Odds ratio (OR) and 95% CI for all meta-analyses carried out. Values in bold are statistically significant.

| Outcome | Subgroup analysis | RCT OR [95% CI]; (N. Studies) | Analytical OR [95% CI]; (N. Studies) |
|---|---|---|---|
| Mortality | **All** | 0.80 [0.61-1.04] (19) | **0.45 [0.24-0.86] (7)** |
| | **Enrollment period** | | |
| | Feb 2020 - May 2020 | **0.32 [0.11-0.91] (2)** | 0.52 [0.19-1.43] (4) |
| | Other | 0.85 [0.64-1.13] (17) | **0.41 [0.17-0.99] (3)** |
| | **Covid-19 Severity** | | |
| | Severe Covid-19 | **0.50 [0.31-0.82] (7)** | 0.65 [0.10-4.19] (2) |
| | Not Severe Covid-19 | 0.99 [0.71-1.38] (12) | **0.41 [0.21-0.77] (5)** |
| | **Age** | | |
| | ≤ 65 years | 1.05 [0.73-1.53] (9) | NA (1) |
| | > 65 years | **0.58 [0.39-0.86] (10)** | **0.48 [0.24-0.95] (6)** |
| | **Baseline Vitamin D status** | | |
| | < 20 ng/ml | 0.56 [0.12-2.67] (3) | 0.63 [0.36-1.09] (3) |
| | Not reported | **0.39 [0.17-0.90] (5)** | NA (1) |
| | Other | 0.95 [0.69-1.31] (11) | 0.48 [0.13-1.72] (3) |
| | **Supplementation** | | |
| | High dosage vs Low | 0.72 [0.42-1.23] (5) | NA |
| | Vitamin D vs No Treat/Placebo | 0.82 [0.60-1.13] (14) | **0.45 [0.24-0.86] (7)** |
| | **Country** | | |
| | Europe | 0.65 [0.39-1.09] (10) | 0.39 [0.15-1.05] (4) |
| | Africa | NA (1) | NA (1) |
| | Asia | 0.63 [0.32-1.22] (3) | 0.59 [0.32-1.07] (2) |
| | America | 1.24 [0.67-2.32] (5) | NA |
| ICU admission | **All** | **0.55 [0.37-0.79] (14)** | **0.35 [0.18-0.66] (5)** |
| | **Enrollment period** | | |
| | Feb 2020 - May 2020 | NA | **0.26 [0.11-0.60] (3)** |
| | Other | **0.55 [0.37-0.79] (14)** | 0.56 [0.29-1.10] (2) |
| | **Covid-19 Severity** | | |
| | Severe Covid-19 | 0.22 [0.02-2.07] (3) | **0.26 [0.11-0.60] (3)** |
| | Not Severe Covid-19 | **0.67 [0.51-0.88] (11)** | 0.56 [0.29-1.10] (2) |
| | **Age** | | |
| | ≤ 65 years | **0.56 [0.32-0.98] (8)** | NA (1) |
| | > 65 years | **0.43 [0.26-0.71] (6)** | **0.29 [0.14-0.58] (4)** |
| | **Baseline Vitamin D status** | | |
| | < 20 ng/ml | 0.59 [0.23-1.51] (3) | NA (1) |
| | Not reported | 0.20 [0.03-1.15] (3) | NA (1) |
| | Other | 0.76 [0.56-1.04] (8) | **0.31 [0.14-0.69] (3)** |
| | **Supplementation** | | |
| | High dosage vs Low | **0.37 [0.20-0.68] (4)** | NA |
| | Vitamin D vs No Treat/Placebo | **0.62 [0.40-0.94] (10)** | **0.35 [0.18-0.66] (5)** |
| | **Country** | | |
| | Europe | 0.37 [0.12-1.08] (6) | **0.31 [0.14-0.69] (3)** |
| | Africa | NA (1) | NA |
| | Asia | 0.46 [0.19-1.11] (3) | 0.42 [0.12-1.49] (2) |
| | America | 0.66 [0.41-1.06] (4) | NA |

| | | | |
|---|---|---|---|
| Intubation | **All** | **0.50 [0.27-0.92] (9)** | 0.65 [0.39-1.08] (3) |
| | **Enrollment period** | | |
| | Feb 2020 - May 2020 | NA | 0.67 [0.29-1.57] (2) |
| | Other | **0.50 [0.27-0.92] (9)** | NA (1) |
| | **Covid-19 Severity** | | |
| | Severe Covid-19 | 1.33 [0.29-6.12] (2) | NA |
| | Not Severe Covid-19 | **0.36 [0.19-0.68] (7)** | 0.65 [0.39-1.08] (3) |
| | **Age** | | |
| | ≤ 65 years | 0.60 [0.37-0.99] (5) | NA |
| | > 65 years | 0.53 [0.10-2.74] (4) | 0.65 [0.39-1.08] (3) |
| | **Baseline Vitamin D status** | | |
| | < 20 ng/ml | 1.55 [0.12-19.17] (2) | 0.65 [0.37-1.13] (2) |
| | Not reported | **0.17 [0.08-0.37] (2)** | NA (1) |
| | Other | 0.62 [0.38-1.02] (5) | NA |
| | **Supplementation** | | |
| | High dosage vs Low | 0.24 [0.06-1.00] (2) | NA |
| | Vitamin D vs No Treat/Placebo | 0.66 [0.41-1.04] (7) | 0.65 [0.39-1.08] (3) |
| | **Country** | | |
| | Europe | 2.59 [0.49-13.62] (2) | NA |
| | Africa | NA (1) | NA |
| | Asia | 0.69 [0.33-1.41] (2) | NA |
| | America | **0.51 [0.26-0.98] (4)** | NA |

**Table S4**. Mean Difference (MD) and 95% CI for all meta-analyses carried out. Values in bold are statistically significant.

| **Outcome** | **Subgroup analysis** | **RCT** Mean Diff. [95% CI]; (N. Studies) |
|---|---|---|
| Hospital Length of Stay | **All** | -0.62 [-1.41, 0.18] (16) |
| | **Enrollment period** | |
| | Feb 2020 - May 2020 | NA |
| | Other | -0.62 [-1.41, 0.18] (16) |
| | **Covid-19 Severity** | |
| | Severe Covid-19 | 2.59 [-0.90, 6.08] (3) |
| | Not Severe Covid-19 | **-0.95 [-1.69, -0.21] (13)** |
| | **Age** | |
| | ≤ 65 years | -0.29 [-0.90, 0.32] (10) |
| | > 65 years | -1.54 [-1.42, 0.18] (6) |
| | **Baseline Vitamin D status** | |
| | < 20 ng/ml | 0.47 [-2.13, 3.06] (2) |
| | Not reported | **-2.93 [-4.39, -1.46] (2)** |
| | Other | -0.41 [-1.19, 0.36] (12) |
| | **Supplementation** | |
| | High dosage vs Low | -0.95 [-2.98, 1.08] (3) |
| | Vitamin D vs No Treat/Placebo | -0.53 [-1.44, 0.38] (13) |
| | **Country** | |
| | Europe | -0.55 [-2.36, 2.26] (5) |
| | Africa | NA (1) |
| | Asia | 0.57 [-0.98, 2.12] (5) |
| | America | **-1.10 [-1.72, -0.47] (5)** |
