# Supplementary figures and images for: The Effect of Vitamin D Supplementation Post COVID-19 Infection and Related Outcomes: A Systematic Review and Meta-Analysis
Source: Nutrients. 2024 Nov 5;16(22):3794. doi: 10.3390/nu16223794 (PMC11597733; doi:10.3390/nu16223794)

**Figure S1.** Summary plot of risk of bias for analytical studies (a) and RCT studies (b).

a

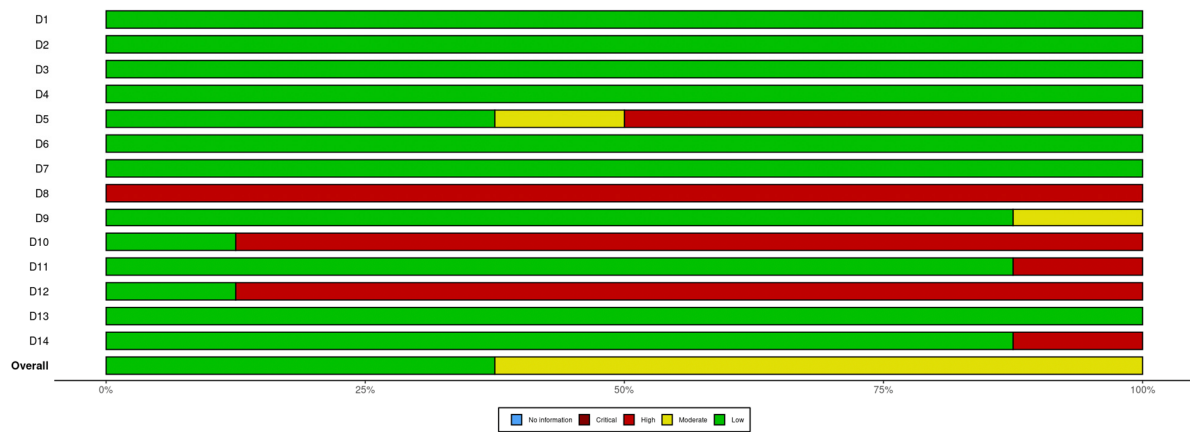

b

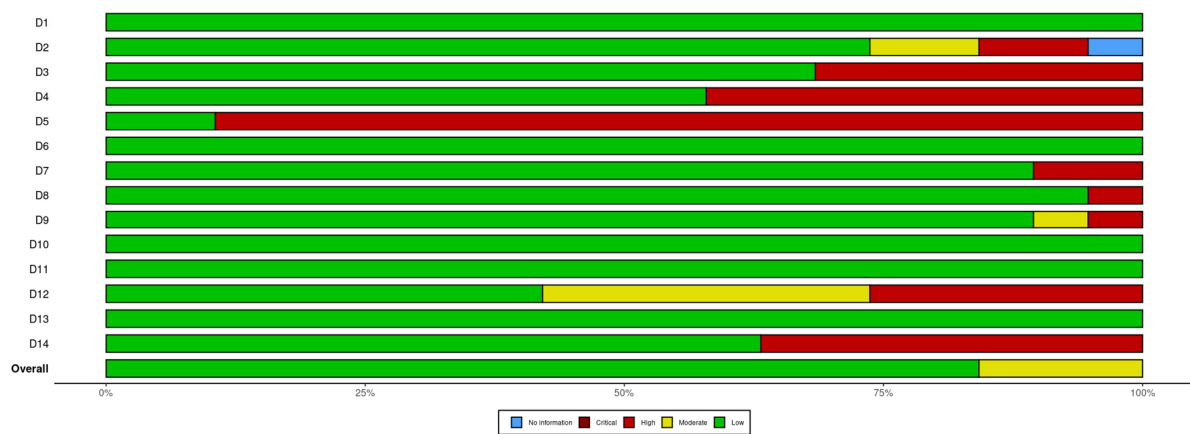

Supplement: Supplementary file 1 [file nutrients-16-03794-s001.zip › Figure S1 a-b Summary plot of risk of bias.pdf]
